# Cardiogeneticsbank@UZA: A Collection of DNA, Tissues, and Cell Lines as a Translational Tool

**DOI:** 10.3389/fmed.2019.00198

**Published:** 2019-09-06

**Authors:** Maaike Alaerts, Gerarda van de Beek, Ilse Luyckx, Josephina Meester, Dorien Schepers, Aline Verstraeten, Johan Saenen, Emeline Van Craenenbroeck, Inge Goovaerts, Inez Rodrigus, Steven Laga, Jeroen Hendriks, Sofie Goethals, Annemieke De Wilde, Elke Smits, Philippe Jorens, Manon Huizing, Lut Van Laer, Bart Loeys

**Affiliations:** ^1^Center of Medical Genetics, Antwerp University Hospital, University of Antwerp, Antwerp, Belgium; ^2^Department of Cardiology, Antwerp University Hospital, University of Antwerp, Antwerp, Belgium; ^3^Department of Cardiac Surgery, Antwerp University Hospital, University of Antwerp, Antwerp, Belgium; ^4^Department of Thoracic and Vascular Surgery, Antwerp University Hospital, University of Antwerp, Antwerp, Belgium; ^5^Biobank, Antwerp University Hospital, University of Antwerp, Antwerp, Belgium; ^6^Intensive Care Unit and Clinical Research Center, Antwerp University Hospital, University of Antwerp, Antwerp, Belgium

**Keywords:** cardiogenetics, biobank, sudden cardiac arrest, inherited cardiac arrhythmia, aortic aneurysm, cardiomyopathies

## Abstract

Cardiogeneticsbank@UZA is an academic hospital integrated biobank that collects aortic tissue, blood, cell lines (fibroblasts, vascular smooth muscle cells, peripheral blood mononuclear cells, and induced pluripotent stem cells), and DNA from patients with cardiogenetic disorders, for both diagnostic and research purposes. We adhere to a quality management system and have established standard protocols for the sampling and processing of all cardiogenetic patient related materials. Cardiogeneticsbank@UZA is embedded in the Biobanking and Biomolecular Resources Research Infrastructure Belgium (BBMRI.be) and samples from this biobank are available for commercial and academic researchers, through an established access procedure. Currently, the extremely valuable cardiogenetics collection consists of more than 8,700 DNA samples, 380 tissue samples, and 500 cell lines of 7,578 patients, and is linked with extensive clinical data. Some interesting potential research applications are discussed.

## Introduction

In 2010 the Cardiogenetics Research Laboratory at the Center for Medical Genetics (CMG) of the University of Antwerp (UA) was founded by Profs. Van Laer and Loeys. This research group focuses on the genetics of thoracic aortic aneurysm and dissection (TAAD), inherited cardiac arrhythmias (Primary Electrical Disease, PED), cardiomyopathies (CM) and hereditary hypercholesterolemia (HC), and the molecular pathophysiological mechanisms underlying these disorders. Over the last years, dozens of genes have been identified as the molecular cause of these cardiogenetic diseases. The research laboratory is closely linked to the Molecular Diagnostic Unit of the CMG. Through a research-diagnostic collaboration, specific next-generation sequencing (NGS)-based molecular diagnostic gene panels for TAAD, PED, CM, and HC were designed and implemented (1, 2). In parallel, Prof. Dr. Loeys established a Cardiogenetics Clinic in collaboration with the Cardiology Department of the Antwerp University Hospital (UZA). With a multidisciplinary team including a geneticist, cardiologists, a genetic counselor, a nurse, and a psychologist, more than 750 consultations of cardiogenetic patients and their family members are performed each year.

The aim of the cardiogeneticsbank@UZA is to systematically collect, store, and distribute different types of samples obtained from cardiogenetic patients or family members for diagnostic and/or research purposes. Initial diagnostic testing mainly involves the NGS-based TAAD, PED, CM, and HC gene panels and/or whole exome sequencing (WES) to identify causal genetic variants. This allows subsequent family testing and counseling, tailored patient management, and potential pre-implantation genetic diagnostics. The current cardiogenetics research projects aim to identify novel genes and genetic modifiers for TAAD, PED, and CM, and analyze the functional effects of the detected variants at molecular, cell, and organ level using cellular and animal models, such as mouse and zebrafish.

The specific disorders covered by the cardiogenetics@UZA database include Brugada syndrome, sick sinus syndrome, Long and Short QT syndrome, Arrhythmogenic Right Ventricular Cardiomyopathy, Catecholaminergic Polymorphic Ventricular Tachycardia, non-ischemic dilated, hypertrophic, non-compaction cardiomyopathy, Marfan syndrome, Loeys-Dietz syndrome, familial thoracic aortic aneurysm/dissection syndrome, bicuspid aortic valve associated aortopathy, vascular Ehlers-Danlos syndrome.

The Cardiogeneticsbank@UZA is part of the larger UZA biobank that was founded with the support of the Flemish initiative for biobanking (CMI) and is now included in the Biobanking and Biomolecular Resources Research Infrastructure network Belgium (BBMRI.be). In the Flemish initiative, different thematic fields were identified, including (auto)immune diseases, infectious diseases, cardiovascular diseases, metabolic diseases and diabetes, neurosciences, oncology, aging, reproductive medicine, and rare disorders. Within the cardiovascular theme, focus on sudden cardiac death (3) was defined and coordinated by the UZA. The sample types include aortic wall and aortic valve tissue, blood, DNA, RNA, skin and vascular fibroblasts, peripheral blood mononuclear cells (PBMCs), and induced pluripotent stem cells (iPSCs). All samples are collected and processed in a standardized way according to the “Standard Operating Procedure” (SOP) protocols stored in the Electronic Lab Notebook (ELN) account of the research group (E-Notebook 2014 Client, version 13.9.0.0, PerkinElmer) or in the document management system DocBase (Acanthis) of the UZA. These SOPs and any updates are validated by a senior scientist and receive a version number and date for correct referencing. All research group members (postdocs, PhD students and lab technicians) are properly trained to follow the correct SOPs and refer to the used protocols correctly in their personal ELNs.

Design and aim of the cardiogenetics@UZA biobank are comparable to other international biobanks focused on cardiovascular disease such as the Cardiovascular Biomedical Research Unit Biobank at the Royal Brompton & Harefield NHS Trust in London (https://www.hra.nhs.uk/planning-and-improving-research/application-summaries/research-summaries/cardiovascular-biomedical-research-unit-biobank/), the GENCOR (4) and CONCOR (5) databases in the Netherlands. Although the latter is mostly focused on structural congenital heart disease.

## Materials and Methods

### Ethics—Informed Consent

For all research samples collected for the biobank, the informed consent corresponding to the correct research project is obtained from the patients, stored in folders at the CMG or electronically stored in the UZA Electronic Patient File (EPD). Every research project has been approved by the local UZA research ethics committee, including the information sheet and consent form for the patients. Patients that attend the Cardiogenetics clinic for diagnostic purposes are properly counseled and informed about potential inclusion in cardiogenetics research projects. They can then provide oral or written consent. If they don't consent, samples are only used for diagnostic purposes.

### Database

All patient identification, clinical, and (genetic) diagnostic data are collected and stored in electronic patient records in the secured Hospital Information Database of the UZA (Joint Commission International (JCI) quality approval, 2017). To this purpose, every patient receives a unique UZA patient identification number (UZA ID). Patients and relatives belonging to the same family receive a unique family identifier and the pedigree is drawn using specific software (PASS). All relevant clinical and diagnostic patient data with corresponding UZA ID and family identifier are then transferred to the Cardiogenetics Research Database (Microsoft Access), stored on a secured UA server with automatic backup. The sample location, date of collection or storage and all research data, including genetic sequence data, functional experiment data and analysis results, are added to this database. Only the Principal Investigator and Medical Doctor Prof. Dr. Loeys can connect the UZA IDs with patient identification data. Hence, for all other research group members the data is anonymized. The necessary GDPR (General Data Protection Regulation) forms for the different data elements gathered in the Cardiogenetics Research Database have been completed and can be provided to the Privacy Commission of Belgium when asked for, in line with recommendation 06/2017 of June 14th 2017 of this commission. All requirements for the EU GDPR (2016/679) have been fulfilled.

### Sample Collection

Blood samples are drawn at the UZA by trained hospital staff. Blood is collected in EDTA-tubes (5 ml) for DNA extraction or PBMC collection, or in PAXgene tubes (Qiagen) for RNA extraction. Aortic wall and valve tissue samples are collected at the UZA operating room and either snap-frozen and stored at −80°C at the UZA Pathology department within 30 min or transferred to the CMG in physiological solution. Skin biopsies are collected at the UZA dermatology department in sterile plastic tubes (Eppendorf) with physiological solution. These samples are transferred to the CMG at room temperature within 24 h after collection, where they are processed or stored at −80°C immediately upon arrival.

### Sample Processing

DNA extractions from blood are performed on an automated nucleic acid extraction system (Perkin Elmer) with robotic liquid handling and DNA is stored at 4° or −20°C after measuring the concentration. RNA extractions are performed using the RNeasy Mini kit (QIAGEN) or the Quick-RNA MiniPrep kit (BaseClear) according to the manufacturer's instructions. RNA is stored in a −80° freezer after measuring the concentration. PBMCs are isolated from blood using a standard protocol based on Lymphocyte Separation Medium and centrifugation steps. The freshly isolated PBMCs are then cryopreserved in liquid nitrogen in a 10% DMSO solution (>3 million cells per cryotube) until further use. Fresh aortic wall or valve tissue samples or skin biopsies are cut in small pieces and digested with trypsin and collagenase, followed by standard culture in fibroblast medium (RPMI medium, Gibco) to obtain vascular, valvular, and dermal fibroblasts, respectively. After culture and expansion these fibroblasts are cryopreserved in liquid nitrogen in a 10% DMSO solution (>2 million cells per cryotube) until further use. Fresh aortic tissue samples are also frozen as a whole at −80°C. For selected patients, vascular smooth muscle cell (VSMC) lines are derived from the fresh aortic wall tissue before cryopreservation.

### iPSC Generation

Both PBMCs and dermal fibroblasts are used to generate patient-specific iPSCs. Thawed PBMCs are cultured in StemSpan medium (STEMCELL Technologies) for 9 days to promote the expansion of hematopoietic cells. Thawed fibroblasts are cultured in regular fibroblast medium until they reach 90% confluency. Next, the Cytotune-iPS 2.0 Sendai reprogramming kit (ThermoFisher Scientific), containing the four Yamanaka transcription factors in non-integrating Sendai viral vectors, is used for the generation of iPSCs, following the manufacturer's instructions. After emergence of iPSC colonies, five rounds of manual picking are performed, followed by five rounds of enzymatic passaging and expansion. At least 12 different clones are selected for cryopreservation based on morphology and growth characteristics and frozen in liquid nitrogen in a 10% DMSO solution. Three of these clones are then fully validated by immunocytochemistry staining for pluripotency markers (Oct4, Nanog, Tra-1-60, and Tra-1-81) and by proving their trilineage differentiation potential using an embryoid body formation assay followed by qPCR assays for endodermal, mesodermal, and ectodermal markers.

### Quality Assurance Measures

DNA and RNA extractions are quality controlled by spectrophotometry-based methods (NanoDrop or Qubit—Thermo Fisher Scientific). The temperature of fridges and freezers is continuously monitored and an alarm system will be activated if the temperature exceeds a specific threshold. The liquid nitrogen tanks are also equipped with an alarm system. All cell cultures are routinely checked to exclude Mycoplasma infection, and once more specifically before cryopreservation. Sustainability of the cardiogenetics@UZA biobank is guaranteed by its embedding within the Antwerp University Hospital and samples are stored for at least 30 years.

### Specimen Types and Numbers

DNA samples: 8,700
RNA samples: 246
Blood samples (unprocessed): 450
Aortic tissue samples: 380
PBMC samples: 55
Fibroblast cell lines: 429—total of 1,860 cryotubes
VSMC lines: 64—total of 130 cryotubes
iPSC lines: 12—total of 610 cryotubes

These samples have been collected from 7,578 patients.

### Access Procedures

Both academic and commercial researchers can be granted access to our collection of samples in the context of a specific collaboration. They will have to submit a Material Request Form in which they describe their project including study design, samples requested, project funding, and scientific relevance. Positive evaluation of this request by the Principal Investigator Prof. Dr. Loeys and the local UZA research ethical committee will lead to the signing of a human Material Transfer Agreement (MTA) and a contract agreeing on the costs to cover the consumables needed for collection, handling and storage of the samples. Samples and their associated coded data can then be released and according to the terms of the MTA the researches are committed to give feedback on sample quality and results and should acknowledge Cardiogeneticsbank@UZA in any scientific communication related to their findings.

## Application Potential

The application potential of a cardiogenetics biobank is extremely diverse but some examples of current applications are discussed below. First, a large collection of DNA samples of patients with well-defined phenotyping can easily be used as a replication cohort for novel candidate genes of cardiogenetic disorders. For many subgroups of cardiogenetic diseases, e.g., dilated cardiomyopathy, Brugada syndrome or thoracic aortic aneurysm (6), the diagnostic mutation yield is far from complete and upon discovery of novel causal genes, validation of these genes can be obtained by resequencing of previously genetically unsolved DNA samples. Second, the collection of region-specific samples (e.g., Flanders) offers the opportunity to identify recurrent mutations in the same gene. Haplotyping can then be performed and identical haplotypes point to Flemish founder mutations. This can initiate larger cascade mutations screening efforts to identify region-specific *at-risk* individuals. At present, we have identified three novel founder mutations in our cardiogenetics biobank. Third, the identification of founder mutations sets the unique platform for the execution of modifier studies. Upon phenotypical characterization of all available founder mutation carriers for a specific gene and condition, one can take advantage of the shared genetic background to identify differences between mutation carriers at the extreme ends of the phenotypical spectrum: e.g., old unaffected (completely asymptomatic) mutation carriers vs. very young affected, clearly symptomatic mutation carriers. Fourth, the collection of aortic wall and valve tissues offers opportunities to study expression patterns both at protein and mRNA level. Finally, the PBMC and fibroblast cultures allow the establishment of iPSC lines, which can be differentiated in cardiomyocytes or vascular smooth muscle cells. As such, these “adult” cell types can be generated from patients with well-defined cardiogenetic disorders for whom the collection of native cardiomyocytes or vascular smooth muscle cells is not feasible. The application potential of these iPSC-derived cell lines is tremendous, as they can be used both for pathomechanistic studies as well as for pharmacological research where they serve as a platform for testing of new drug compounds.

In conclusion, using well-documented standard operating procedures and quality control, combined with excellent and detailed clinical data from an extended network, the Cardiogeneticsbank@UZA provides an extremely valuable collection of patient samples that is used for both diagnostic and research purposes.

## Data Availability

The datasets generated for this study are available on request to the corresponding author.

## Author Contributions

MA, GvdB, LV, and BL drafted the paper. IL, JM, DS, AV, JS, EV, IG, IR, SL, JH, SG, AD, ES, PJ, and MH have revised the paper. All co-authors have contributed to the establishment and sample collection for the cardiogenetics@uza biobank.

### Conflict of Interest Statement

The authors declare that the research was conducted in the absence of any commercial or financial relationships that could be construed as a potential conflict of interest.
